# Accuracy of death certification and hospital record linkage for identification of incident stroke

**DOI:** 10.1186/1471-2288-8-74

**Published:** 2008-11-10

**Authors:** Shubhada Sinha, Phyo K Myint, Robert N Luben, Kay-Tee Khaw

**Affiliations:** 1 West Suffolk Hospital, Hardwick Lane, Bury St Edmunds, IP33 2QZ, Suffolk, UK; 2 Ageing and Stroke Medicine Section, Population Health Group, School of Medicine, Health Policy and Practice, University of East Anglia, Norwich, NR4 7TJ, Norfolk, UK; 3 Strangeways Research Laboratory, Department of Public Health and Primary Care, University of Cambridge, Worts Causeway, Cambridge, CB1 8RN, UK

## Abstract

**Background:**

There is little information on the validity of using record linkage with routinely collected data for case ascertainment of stroke in large population-based studies in the UK. We examined the accuracy of these routine record linkage approaches for identifying incident stroke cases in a large UK population-based study, the European Prospective Investigation into Cancer (EPIC)-Norfolk cohort.

**Methods:**

We examined a sample of hospital records of incident stroke cases identified by linkage with two routine data sources, death certificates and a national hospital record linkage system (ENCORE), using predefined study criteria. Two senior Specialist Registrars with clinical experience in stroke medicine examined the hospital records and searched for the evidence of stroke recorded in these records between 1993/97–2003.

**Results:**

Of 520 incident strokes identified between 1993/1997–2003 using record linkage systems in the EPIC-Norfolk, a sample of 250 medical case notes were examined between March and July 2004. Using the predefined study criteria, there were 191 definite strokes (76%), 20 probable strokes (8%), 11 possible strokes and 11 cases of transient ischaemic attacks (4% each) i.e. 233/250 (93%) with possible or definite stroke or transient ischaemic attacks. Stroke could not be verified using hospital records in 13 cases (5%) and 4 cases (2%) had other diagnoses: 3 cases of vascular dementia and 1 case of benign intracranial hypertension. The diagnosis of stroke in 185 out of 250 cases identified in the EPIC-Norfolk (74.0%) was supported by radiological evidence using WHO criteria.

**Conclusion:**

Death certificates and hospital record linkage in this British prospective study have a high accuracy or positive predictive value in correctly identifying incident stroke cases.

## Background

Deeper insight and better understanding of stroke epidemiology plays an important role in its primary prevention. Large population-based studies with long-term follow-up provide valuable information on prospective relationships between biological, physiological and lifestyle factors and the risk of incident stroke. Most of them rely on self-report and/or death certification data for case ascertainment. Previous validation studies of self-reported prevalent and incident stroke showed high percentages of both false positive and false negative cases that varied between different studies [1-9].

Death certification data are routinely collected in most developed countries. Use of such data has the advantage of the potential for completeness of follow up in participants of prospective cohort studies but this approach may identify only a limited proportion of cases of stroke as many incident events are non-fatal. Self report, followed by retrieval and validation of medical records has the disadvantage of incomplete ascertainment due to drop out and low response rates to follow-up questionnaires. Underreporting from participants who continue to participate in the study is also an issue. In the United Kingdom, the National Health Service provides the potential for identifying all hospital admissions by condition for individuals in Britain and therefore the potential for more complete ascertainment of stroke using hospital record linkage.

In this study, we determined the proportion of strokes identified using death certificates and routine hospital record linkage in a prospective population study that could be confirmed as strokes based on clinical information from the medical notes. We also examined the proportion of confirmed stroke subtype (ischaemic or haemorrhagic) based on information obtained from the medical notes.

## Methods

The European Prospective Investigation into Cancer (EPIC)-Norfolk is a population-based study with more than 30,000 participants. It included ~40% of the free-living population from selected general practices in Norfolk, UK between the ages of 40–79 at the baseline. The general aims were to understand the determinants of chronic disease. In addition to regular self-administered postal questionnaires, participants are followed up through record linkage with two routinely collected data sources: death certification and hospital record linkage. The Norwich Local Research Ethics Committee approved the study. We have previously reported the prospective relationships between lifestyle and biologic factors and stroke in the EPIC-Norfolk cohort with stroke identified using death certificates and hospital record linkage data (ENCORE – **E**ast **N**orfolk **CO**mmission **RE**cord.) [10-15]. ENCORE is the database of hospital admissions nationally on Norfolk residents kept by the East Norfolk Health Commission as part of the National Health Service.

We conducted a validation study for stroke cases identified from the EPIC-Norfolk population cohort study using the two record linkage methods up to a follow-up date of 31^st ^March 2003. We examined hospital medical records of a subset of approximately 50% of those who were identified as having incident stroke between the participant's enrolment into the study at the baseline (1993–1997) and the follow up date in 2003 who had available records from one main hospital in Norfolk. Incident strokes were defined as those cases of stroke who were free of stroke at the time of enrolment to the study but identified as having stroke by two record linkage methods, death certificates from Office of National Statistics (ONS) and a hospital record linkage system, ENCORE during the follow-up period. The hospital case notes were retrieved from the medical health records department of the Norfolk and Norwich Hospital between March-July 2004.

In the EPIC-Norfolk study, prevalent strokes were identified by response to the question "*Has a doctor ever told you that you have any of the following*" followed by a list of chronic medical conditions including stroke at the baseline health survey. Incident strokes were identified using 2 methods. The first method used data available from the the death certificates when; stroke is mentioned as primary cause of death (i.e. I a, b, c) or conditions which may contribute to death (II). All participants have been flagged for death certification at the UK office of National Statistics (ONS), with vital status ascertained for the whole cohort.

The second method used ENCORE (**E**ast **N**orfolk **CO**mmission **RE**cord), the database of hospital admissions kept by the East Norfolk Health Commission as described above. ENCORE provides data on admissions to hospital for all except those who are admitted to private hospitals which are minimal in Norfolk for serious acute conditions such as stroke.

We also sent two follow-up health questionnaires 18 months after baseline and between 2000–2004 which included questions about incident strokes but these data are not included in the current analyses. ICD 10 (the Tenth revision of the International Classification of Diseases) coding I60–I69 was used to identify strokes from death certification and hospital admission records.

The Norfolk and Norwich University Hospital serves the city of Norwich and surrounding rural areas, from which the EPIC-Norfolk study population was recruited and 97% of all hospital admissions in Britain of the EPIC-Norfolk participants identified through record linkage are in the Norfolk and Norwich Hospital group. Of the 520 strokes identified using record linkage, we retrieved case notes from the medical records department of Norfolk and Norwich University Hospital for a sample of 250 people. Two of the investigators (SS and PKM), then senior Specialist Registrars/Stroke Clinical Research Fellows, examined the hospital records for the evidence of incident stroke using a standardised data collection form (Additional file 1) between March and July 2004.

For this study purpose, we pre-defined "incident stroke" using criteria based on the current WHO clinical definition [16]. It defines stroke as "*sudden onset of focal/global neurological deficit lasting more than 24 hours due to presumed vascular aetiology*". Focal neurological signs considered to be of significance in the current study include any objective locomotor or sensory signs, dysphasia, visual field loss without other explanation, apraxias and other perceptual deficits, and ataxia. Global signs include "coma" but exclude isolated changes in cognitive function and transient global amnesia.

We categorised the patients into following three categories.

(1) definite stroke when there was a documented definite focal neurology over 24 hours or persisted focal neurological deficit till death in those who died within 24 hours (or) probable stroke with supporting evidence on CT or post-mortem examination

(2) probable stroke when it was unclear whether focal neurological deficit lasted > 24 hours or neurological signs were doubtful (i.e. when clinician who saw the patient at the time was unsure of significance of physical signs – e.g. apparent unilateral weakness in an unwell Parkinson's Disease patient) or global (e.g. reduced conscious level where focal neurological signs are difficult to elicit)

(3) possible stroke when there was mention of stroke in hospital medical records (e.g. past medical history or in the GP referral letter or correspondence between primary and secondary care which has occurred after 1997) with no supporting clinical information available in the medical entry.

## Results

There were a total of 520 incident strokes identified by the death certification and hospital record linkage. Inclusion of self report further increased the number of incident strokes to a total of 726. Out of 520 incident strokes identified in the EPIC-Norfolk between 1993/1997–2003 using death certificates and ENCORE, we examined 250 medical case notes identified from one main hospital, the Norfolk and Norwich, during the study period. Out of 520 incident strokes, 176 were confirmed by hospital records and 423 by death certificates. Only 250 sets of case notes were retrieved for logistical reasons. The reason being the case notes were not available in the Health Records Department at the time of request for note retrieval. The reason for them not being available is due to these case notes being in other hospital departments rather than medical records.

Using the pre-defined study criteria mentioned above, there were 191 definite strokes, 20 probable strokes and 11 possible strokes. There were further 11 cases where neurological deficit lasted < 24 hours which were classified as transient ischaemic attacks (TIAs) as per current WHO criteria. There was no evidence of stroke in Norfolk and Norwich University Hospital case records in 13 cases and there were 4 cases with other diagnoses. There were 3 cases of vascular dementia with the CT evidence of cerebrovascular disease and a case of benign intracranial hypertension.

Out of 250 cases validated, 185 had a computed tomography (CT) scan and/or magnetic resonance imaging (MRI) during their hospital admission. Of those who had a neuro-imaging study, 144 were confirmed ischaemic strokes (cerebral infarct or radiological evidence of cerebrovascular disease), 22 were confirmed primary intracerebral haemorrhage. There were 14 cases of subarachnoid haemorrhage and 6 cases of subdural haematoma.

The results were summarised in the table 1. The table 1 also shows the respective proportions (in percent) of correctly identified strokes by various clinical criteria.

**Table 1:** Number and percentage of 250 strokes identified through linkage with routine death certification and hospital records confirmed by hospital clinical record inspection in EPIC Norfolk 1993–2003

Method	250 strokes identified in EPIC-Norfolk using routine record linkage	
Study criteria (Additional file 1)	Number	Percent
(a) = Definite stroke	191	76.4
(b) = (a) + 20 Probable stroke	211	84.0
(c) = (b) + 11 Possible strokes	222	88.8
(d) = (c) + 11 TIAs	233	93.2
(e) = (d) + 3 vascular dementia	236	94.4

## Discussion

In the current study 222 out of 250 cases had definite, probable or possible stroke as per pre-defined study criteria. Three out of four other cases were in fact vascular dementia in those who had no previous history of stroke and therefore had evidence of incident cerebrovascular disease. Thus, 225 out of 250 and these could be regarded as true incident strokes. Eleven cases in this study were classified as TIA by the investigators as per current WHO criteria. Most of them, however, were highly likely to have high risk of developing stroke and in fact some of them might have been mild strokes which resolved within 24 hours. In any case these 11 cases were likely to carry the same vascular risk as stroke cases and epidemiology of these cases were unlikely to be different from epidemiological perspective in terms of stroke risk identification. Therefore, 236 cases out of 250 cases identified in the EPIC-Norfolk by the death certificates and hospital record linkage had cerebrovascular disease.

In 13 cases we could not find evidence of stroke in hospital records. This may be due to community deaths due to stroke, which would have been captured only in the death certificates but not in the hospital records. Some of them may also had stroke but were not admitted to the Norfolk and Norwich hospital. Therefore, the estimate figures we report may be underestimation of the accuracy of case ascertainment. To a certain extent, the false positivity i.e. identifying non-stroke cases as stroke may be partly explained by the fact that we identified incident stroke cases using ICD 10 (I60–I69) which included subarachnoid hemorrhages and subdural hematomas. The CT scan rate of the cases (185/250, 74.0%) in this study is comparable to the earlier report by Myint et al, which examined the CT scan rate in the Norfolk and Norwich University Hospital [17].

The completeness of hospital admission data for identifying strokes will depend on the proportion of people with stroke who are admitted to hospital. In the Rotterdam Study [4] only 53% of people with confirmed stroke were admitted to hospital. Frequency of hospitalisation varies from 96% in Finland to 55% in UK. However, the caveat is that with increasing awareness of stroke as an emergency condition, hospital admissions for stroke have increased over the last decade in the UK.

Heliövaara and colleagues [18] previously compared hospital discharge data with self-reports, validated using medical records. 80% of self reported strokes were recorded in the hospital discharge registers. Of these, 82% agreed with validated self-report. Strokes unconfirmed from hospital data were 19% of all recorded strokes. The proportion of death and hospital record ascertained strokes not subsequently confirmable in our study is comparable to their study.

In the MONICA populations, the proportion of fatal strokes not admitted to hospital varied between 0–37%, while the proportion of non-fatal, non-hospitalised strokes compared with all strokes was 0–16% [19]. However, these data are subject to miscoding and errors. In the current study, the 13 cases identified through record linkage which we could not verify with evidence of incident stroke in the hospital clinical records could have been either community deaths, or coding or other errors.

Furthermore, we used ICD 10 coding (I60–I69) to identify incident strokes to be comparable with other epidemiological studies of this scale and it has been acknowledged that using ICD coding has potential weakness [20].

Iso et al [21] examined records of in-hospital deaths coded as stroke in participants in the Minnesota Heart Study aged 30–74 in 1980. The positive predictive value (PPV) of death certificate coding of stroke was 100% (i.e. all cases with death certificates coded as stroke were stroke by study criteria). Sensitivity for stroke was 70% (i.e. 30% of stroke deaths were not coded as such). The PPV of intracranial haemorrhage was 82% and of non-haemorrhagic stroke was 97%. Thus, though death certificates may miss a proportion of stroke deaths, those deaths that are identified as stroke deaths are virtually all correct. In their sample 72% of cases had had either a neuro-imaging study or a necropsy.

In this study, we did not address the question of sensitivity of record linkage methods for identifying strokes, that is, what proportion of true strokes occurring in the population were not identified using death certificate data and hospital record linkage. It is quite likely that some strokes were not admitted to hospital but managed in the community. We did not validate the strokes which were identified by self-report. In population studies on stroke incidence this is one of the most difficult questions: how to find and ascertain those stroke cases who survive their strokes but are not hospitalized. Nevertheless, while stroke incidence is therefore very likely to be under-estimated in epidemiological studies using routine record linkage, since strokes are a relatively rare condition, in large population studies, missed stroke cases in the denominator are unlikely to have an undue effect on analyses examining risk factor associations. More important is whether stroke cases ascertained are indeed likely to be true stroke cases. In this study we have shown that using both death certificate data and hospital record linkage data in large epidemiological studies in the UK setting is highly reliable despite a number of limitations described above with 94% accuracy.

The data were collected by two observers. Although there was a prior agreement between the observers (SS and PKM) with regards to criteria used and how to collect the data for standardisation, we did not test the inter-observer agreement. However, the fact that the care of the patients were provided by the single NHS hospital and patients were managed either by neurologist or geriatricians with interest in stroke medicine limit the variability of type and quality of information obtainable in hospital records. One issue is the generalisability of the Norfolk experience. At the time of the study, there were low rates of neuroimaging and stroke admissions compared to many other countries. There might therefore be some selection bias resulting from mostly major strokes being captured in the study.

Nevertheless, these estimates are likely to be an underestimate of the accuracy of stroke diagnoses ascertained through routine hospital data. With the increasing trend in hospitalisation of strokes and better CT scanning rate [17] it is likely that the completeness and accuracy of stroke ascertainment using record linkage with routine death certification and hospital record linkage system in the UK will improve.

## Conclusion

In conclusion, our results suggest that hospital record linkage provides a feasible and reasonably accurate method of identifying strokes in cohort studies in the UK.

## Abbreviations

ENCORE: East Norfolk Commission Record; EPIC-Norfolk: EuropeanProspective Investigation into Cancer-Norfolk; ICD 10: the 10^th ^Revision of the International Classification of Diseases; NHS: National Health Service; ONS: Office of National Statistics; PPV: Positive Predictive Value; TIA: Transient Ischaemic Attack; WHO: World Health Organisation.

## Competing interests

The authors declare that they have no competing interests.

## Authors' contributions

KTK is a principal investigator in EPIC-Norfolk population study. RNL is responsible for data management, computing and data linkages. SS and PKM reviewed and verified strokes cases by examining hospital records, and prepared the draft manuscript. All co-authors contributed in writing of this paper. KTK is the guarantor.

## Pre-publication history

The pre-publication history for this paper can be accessed here:



## Supplementary Material

Additional file 1 **Appendix.** Proforma for possible stroke event. Click here for file
