# Supplementary material for: Accuracy of death certification and hospital record linkage for identification of incident stroke
Source: BMC Med Res Methodol. 2008 Nov 10;8:74. doi: 10.1186/1471-2288-8-74 (PMC2605452; doi:10.1186/1471-2288-8-74)
Supplement: Additional file 1 — Appendix. Proforma for possible stroke event. [file 1471-2288-8-74-S1.doc]

# *Appendix*

# Proforma for possible stroke event

Name -------------------------------------------------------

Hosp number -------------------------------------------------------

NHS number -------------------------------------------------------

Date of birth

Date of stroke

Admitted to hospital as in-patient Yes/No

**Definite stroke** Definite focal neurology

Over 24 hours or death within 24 hours

**OR** probable stroke with supporting evidence on CT or post-mortem

**Probable stroke** unclear whether lasted > 24 hours

Neurological signs doubtful or global

**Possible stroke** mention of stroke in notes (eg PMH/GP letter) with no supporting clinical information

**No mention of stroke in notes**

**Other diagnosis (state what):**

Clinical features Weakness r l arm leg face

Sensory disturbance r l arm leg face

Visual field loss r l

Aphasia

Apraxia r l

Inattention r l

Ataxia r l central

Coma

Alive on discharge yes/no

Date of discharge/death ______________________

Previous stroke in medical record yes/no

CT scan performed? Yes/no

Date of CT scan

Result of CT scan

Necropsy performed Yes/no

Result of necropsy
